# Brain Structural Correlates of Risk-Taking Behavior and Effects of Peer Influence in Adolescents

**DOI:** 10.1371/journal.pone.0112780

**Published:** 2014-11-12

**Authors:** Myoung Soo Kwon, Victor Vorobyev, Dagfinn Moe, Riitta Parkkola, Heikki Hämäläinen

**Affiliations:** 1 Centre for Cognitive Neuroscience, Department of Psychology, University of Turku, Turku, Finland; 2 Department of Transport Research, SINTEF Technology and Society, Trondheim, Norway; 3 Department of Radiology, University Hospital of Tampere, Tampere, Finland; Institute of Psychology, Chinese Academy of Sciences, China

## Abstract

Adolescents are characterized by impulsive risky behavior, particularly in the presence of peers. We discriminated high and low risk-taking male adolescents aged 18–19 years by assessing their propensity for risky behavior and vulnerability to peer influence with personality tests, and compared structural differences in gray and white matter of the brain with voxel-based morphometry (VBM) and diffusion tensor imaging (DTI), respectively. We also compared the brain structures according to the participants' actual risk-taking behavior in a simulated driving task with two different social conditions making up a peer competition situation. There was a discrepancy between the self-reported personality test results and risky driving behavior (running through an intersection with traffic lights turning yellow, chancing a collision with another vehicle). Comparison between high and low risk-taking adolescents according to personality test results revealed no significant difference in gray matter volume and white matter integrity. However, comparison according to actual risk-taking behavior during task performance revealed significantly higher white matter integrity in the high risk-taking group, suggesting that increased risky behavior during adolescence is not necessarily attributed to the immature brain as conventional wisdom says.

## Introduction

Adolescence is characterized by novelty-seeking and impulsive risky behavior [1], and a stronger motivation for peer acceptance than children and adults [2], [3]. Thus, adolescent risk-taking is much more likely to occur in the presence of peers, as evidenced in reckless driving [4], substance abuse [5], and crime [6]. Recent experimental studies also indicate that adolescents' decisions are directly influenced by the mere presence of peers, showing that they took substantially more risks in behavioral tasks when observed by peers [7], [8].

The human brain undergoes dramatic structural changes during childhood and adolescence, and adolescents' heightened propensity for risky behavior is thought to reflect maturational imbalance between affective reward processing and cognitive control systems [1], [9]–[16]. The reward processing system involving the ventral striatum (including the nucleus accumbens) and the orbitofrontal cortex changes dramatically in early adolescence, while the cognitive control system including the lateral prefrontal and dorsal anterior cingulate cortex undergoes comparatively gradual maturation. Thus, increased sensitivity to rewards, paired with immature cognitive control ability to down-regulate the reward system, may bias adolescents' decisions toward greater risk-taking, and the interaction with peers may further sensitize the reward system to potential rewards of risky behavior.

In support of this ‘dual systems’ theory, a recent functional magnetic resonance imaging (MRI) study [17] showed that the influence of peers on adolescents' decisions was reflected in the increased activation of reward-related brain regions, including the ventral striatum and orbitofrontal cortex. During peer observation, adolescents demonstrated greater activation in reward-related brain regions, and the activity predicted subsequent risk-taking. Brain areas associated with cognitive control were less strongly recruited by adolescents than adults, and the activity did not vary with social context. This suggests that the presence of peers increases adolescent risk-taking by increasing sensitivity to potential rewards of risky decisions.

According to MRI-based morphometry studies, gray matter (GM) and white matter (WM) also show different developmental trajectories. Specifically, GM volume rapidly increases early in development, peaking at around age 4, and decreases thereafter [18]–[21]. The loss of GM during childhood and adolescence reflects a refining process of the immature brain, such as synaptic pruning. In addition, brain regions associated with primary functions such as the sensory and motor cortices mature first, followed by the parietal and temporal association cortices, and then higher-order association areas such as the prefrontal cortex involved in top-down control of behavior [18], [20]–[24] (for a review, see [25]). In contrast, WM volume and density increases steadily in a linear pattern up until young adulthood [18], [20], [21], [26], [27]. These changes reflect ongoing myelination of axons enhancing neuronal conduction and communication. Thus, neural connections in the brain are fine-tuned with the elimination of overabundant synapses in GM and strengthening of relevant connections in WM with development and experiences.

To date, brain imaging research on adolescent risk-taking (and peer influence) has been focused on functional brain activation associated with reward processing and cognitive control, particularly in different age groups (e.g., [17]), while research on brain structural differences or changes in the developing adolescent brain has been relatively rare, and the findings were inconsistent. In some study, for instance, immature WM (with lower fiber integrity) predicted risky behavior such as substance abuse [28], while in other studies, lower WM integrity was associated with greater (not less) impulse control [29], or engagement in risky behavior was associated with greater (not less) WM maturity in the frontal cortex [30]. It should be noted that previous studies mainly used self-report assessment of impulse control [29] and risky behavior [30], and diffusion tensor imaging (DTI) for WM integrity. Regarding GM, voxel-based morphometry (VBM) showed that greater risk-taking preference in a monetary incentive delay (or gambling) task and potential substance abuse was associated with lower GM density in the ventral striatum [31].

In the present study, we compared both GM volume and WM integrity of high and low risk-taking groups of adolescents assessed by personality tests on propensity for risky behavior and vulnerability to peer influence. We also evaluated differences in the brain structure according to the participants' actual risk-taking behavior verified in a simulated driving task with two different social conditions involving peer influence. The social context was manipulated by informing the participants that the performance would be compared among their peers.

## Materials and Methods

### Participants

Adolescent participants were recruited from a local vocational school after assessing their propensity for risky behavior and vulnerability to peer influence with personality tests. A total of 215 students completed the tests administered online in a computer class. Excluding females and left-handers, 187 were further examined for extreme cases on the scales for impulsivity (sensation-seeking) and resistance to peer influence, and 43 high risk-taking (high impulsivity and low resistance to peer influence) and 46 low risk-taking (low impulsivity and high resistance to peer influence) respondents were selected, and 17 from each group agreed to participate in the experiment. We confirmed with histograms and a scatter plot that the scores (n = 187) were normally distributed, and the selected respondents and participants for each group were scattered randomly within its quadrant divided by the average scores. Thus, the participants were 34 right-handed male adolescents aged 18–19 years with no history of neurological or psychiatric problems.

The study was approved by the Ethics Committee of the Hospital District of Southwest Finland, and conducted at the Department of Radiology of the Turku University Hospital (TYKS), Turku, Finland, according to the Declaration of Helsinki. The participants gave written informed consent and received monetary compensation (150 euro) for their participation.

After the experiment, MRI data of some participants were excluded due to failure to follow instructions or problems in data registration. Thus, between-group MRI analyses included 32 participants (16 per group) and correlation analyses between MRI measures and the social factor included 29 participants (16 high and 13 low risk-taking groups).

### Personality tests

We used Zuckerman-Kuhlman-Aluja Personality Questionnaire (ZKA-PQ) [32] and Resistance to Peer Influence Scale (RPIS) [33]. In the ZKA-PQ, each factor consists of four facets with 10 questions each, and we used questionnaire items that represent sensation-seeking (thrill- and adventure-seeking, experience-seeking, disinhibition, boredom susceptibility/impulsivity) and neuroticism (dependency, low self-esteem) factors, resulting in 60 out of 200 items. The two facets of neuroticism were regarded as sensitive to peer influence, while the other two (anxiety, depression) were not included. The RPIS included all ten items. The tests were translated from English into Finnish by a professional translator, and administered online using Webropol 2.0 (Helsinki, Finland).

### Driving task

We used a simple computerized driving task modified from the Stoplight Game [17], in which the participant controls the progression of a vehicle along a straight track, from a driver's point of view. The track consisted of 20 intersections with traffic lights, each treated as a separate trial. As the vehicle approaches the intersection, the traffic light turns yellow and the participant decides whether to stop and wait for a red light to turn green, or to keep driving and chance a possible crash at the intersection. The decision was made by pressing either Go or Stop button after the light turns yellow. The Stop and Go buttons were placed under the index and middle fingers of the right hand, respectively, being on the same side of the brake and accelerator pedals of a car.

The traffic light at the first intersection always remained green, and thus only the other 19 trials were included in the data analysis. Successfully traveling through an intersection without braking (Go) resulted in no delay, whereas braking (Stop) and waiting for the red light to turn green resulted in a short 3-s delay. Unsuccessful traveling with a crash at an intersection results in a longer 6-s delay. Risk-taking (i.e., not braking for the yellow light) was encouraged by instructing to complete the track as quickly as possible.

The game script included varying distance between intersections, or inter-trial interval (11–13 s), and timing of the traffic light turning yellow before entering an intersection (1.5–3.0 s). The probability of the crash at the intersection also varied as another car, invisible in advance, crossed the track either immediately (causing an inevitable crash for Go responses, 7 trials) or 2.0 s (12 trials) after the participant's car arrived at the intersection. Details of the task parameters will be reported separately. Participants were given 5 min to complete the track and the worst case with 12 stops and 7 crashes took 5 min 12 s to reach the end of the track. There were six variants of the game script, two for practice sessions and the other four for the actual experiment, presented in a counterbalanced order across participants.

### Procedure

The participant was first given instructions and performed a short guided game of 10 trials, sitting in a chair outside the MRI room. The participant then performed two full-length practice runs with instructions to reach the end of the track as quickly as possible. The participant was then placed in an MRI scanner and completed four runs of the task under two different social conditions, the first two runs in the non-competition condition and the other two runs in the competition condition. In the non-competition condition, the participant was simply instructed to reach the end of the track as quickly as possible, in the same way as in the practice runs, whereas in the competition condition, they were told that the results of their performance would be presented at school and compared among their peers to reveal the fastest record. The change of social context was a surprise manipulation. Instructions on the social condition were given before starting every run of the task.

It took less than 1 h to complete six runs of the task (including two practice runs), and the whole experimental session including instructions, positioning in the scanner, and structural MRI scans before and after task performance took about 2 h.

### Behavioral data

The number of Go responses (or risk-taking), and Go and Stop response (or decision-making) time were compared between two groups (high and low risk-taking) and two social conditions (competition, non-competition). The behavioral data showed that the personality tests could not predict actual risk-taking behavior in the driving task. The questionnaire and behavioral data will be reported separately. In short, participants took more risks and spent more time in making either Go or Stop decision during the competition condition, without any difference between the questionnaire-based groups. Thus, we also considered performance-based group comparisons for the MRI data using the number of Go responses.

An index of social influence was calculated as a ratio between the number of Go responses registered in the conditions with and without a peer competition situation. This index was used in the GM volume and fractional anisotropy (FA) analyses. Only 29 subjects (out of the 32 used for other analyses) had valid social indices: Two subjects reported after experiment that they did not hear or understood the instruction about a shift to competition, and one subject made no risky decisions during non-competition sessions.

### MRI data acquisition and processing

A set of structural images including T1-weghted MRI and diffusion data set was obtained with the Verio 3T MRI scanner (Siemens Medical Systems, Erlangen, Germany) at the Medical Imaging Centre of Southwest Finland, Turku, Finland. A high-resolution T1-weighed 3D magnetization-prepared rapid gradient-echo (MP-RAGE) [34] scan of the entire brain (TR = 2300 ms, TE = 3.43 ms, TI = 900 ms, flip angle  = 9°; matrix size  = 256×200 mm, voxel size  = 1×1×1 mm) was obtained for morphometric analysis and for calculating spatial normalization parameters in both morphometric and diffusion data processing. The diffusion dataset for each participant included a T2-weighted image with no diffusion gradient (b0 image), and 64 images obtained with diffusion gradients (b = 1000 s/mm^2^) applied in 64 isotropically distributed encoding directions. The diffusion imaging was performed using 7300 ms TR, 92 ms TE, 90° flip angle, 56 axial slices with 2 mm cubic voxel size, and 112×112 mm acquisition matrix. Image processing and statistical analysis was performed with Statistical Parametric Mapping (SPM8; Wellcome Department of Cognitive Neurology, London, UK) [35] and related toolboxes implemented in Matlab 7.4.0 (MathWorks, Natik, MA).

T1-weighted images were segmented into tissue classes of GM, WM, and cerebrospinal fluid (CSF) using the New Segment toolbox in SPM8. Then, the GM and WM images were entered into the diffeomorphic anatomical registration through exponentiated Lie algebra (DARTEL) procedure [36]. This procedure included iterative calculations resulting in creation of a group-specific template and a series of flow-filed images containing parameters of nonlinear warping of individual images to the template. The resulting group template was then normalized to the standard space of the Montreal Neurological Institute (MNI) using affine transformations. After that, all the individual GM images were normalized to the MNI space by using corresponding combinations of a DARTEL flow-field and affine transformations. The Jacobian modulation [37] was performed at this step to perceive local GM volume values in the normalized images. The normalized modulated GM images were smoothed with an 8-mm full width at half maximum (FWHM) Gaussian kernel.

Diffusion-weighed images were corrected for head motion and eddy currents [38]. Each participant's brain mask was obtained by binarizing (threshold voxel value>0.1) individual b0 images skull-striped with the brain extraction tool (BET) implemented in C. Rorden's MRICron (http://www.mccauslandcenter.sc.edu/mricro/mricron/index.html). The diffusion tensor for each voxel was estimated by the ordinary method of least squares implemented in the artefact correction in diffusion MRI (ACID) toolbox for SPM [39]. This step also produced individual images of FA containing voxel-wise rotationally invariant estimates of diffusion anisotropy. The FA images were then spatially normalized into a common space. For this, each FA image was coregistered to a corresponding WM image (obtained at the segmentation step of the T1-weighed images) by maximization of normalized mutual information [40]. Then, the coregistered FA images were normalized to the MNI space by applying the same transformations as in the normalization of GM images, without modulation. Finally, the normalized FA images were smoothed with a Gaussian kernel of 8-mm FWHM.

### Models and statistical analysis

Each type of data (GM volume and FA) was analyzed in three ways, all based on the general linear model as implemented in SPM8. First, between-group differences in high and low risk-taking groups based on either questionnaire or performance data were explored by two-sample t-tests. Second, in order to check if using a continuous regressor, instead of group comparison, could be more sensitive for some activations, an additional model (multiple regression) was used including both total number of Go responses and questionnaire score of risk-taking propensity as covariates. Finally, because only 29 subjects had a valid social influence index (see Behavioral data), a separate model including the index as a single regressor of interest was used to estimate the influence of peer competition.

In the GM volume analysis, each model also included a regressor of individual differences in total intracranial volume (GM + WM + CSF) to account for the global effect and the analysis was restricted by a binary mask that was created by using an optimal threshold [41] to the mean of normalized smoothed GM images. For FA, the analysis was restricted by a volume with voxel values of >0.3 in the mean smoothed normalized FA image.

A cluster-level criterion for significance was used for all obtained local effects in both GM volume and FA. A cluster-defining uncorrected voxel threshold was set at p<0.001 [42]. A cluster-level significance threshold was set at p<0.05 corrected for family-wise error (FWE) rate based on the Gaussian random field theory. Localization of effects was done with Johns Hopkins University (JHU) WM tract labels included in the C. Rorden's MRICron software.

## Results

We found no significant effects in the GM volume analyses with group comparisons (High vs. Low risk-taking group) or correlation using the total number of Go responses.

**Figure 1:**
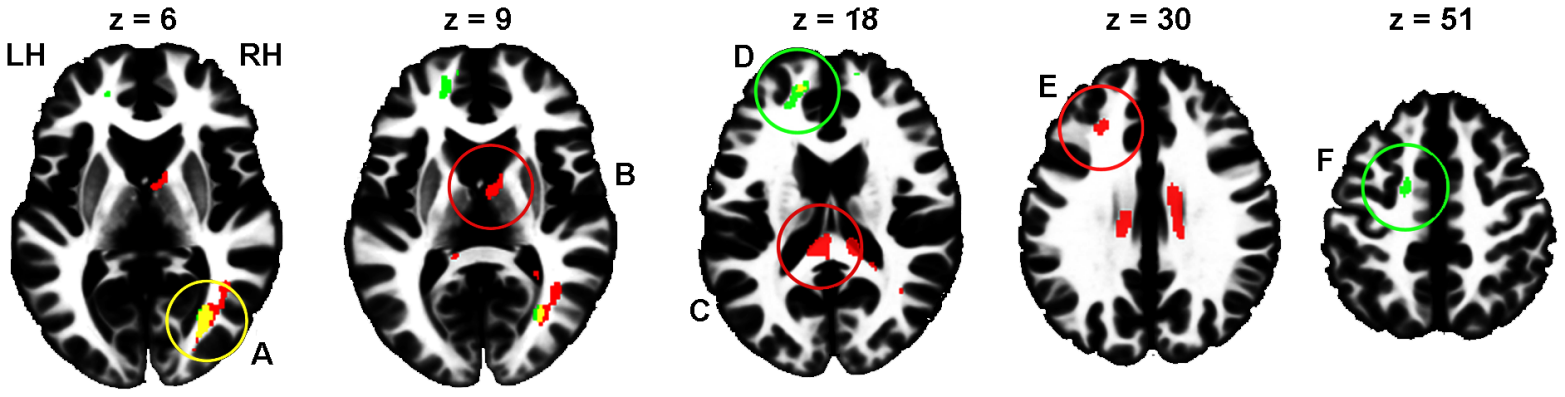
Areas of significant (cluster size p<0.05 after FWE correction) difference between performance-based High and low risk-taking groups (red), areas of positive correlation between the total number of Go responses and FA (green), and overlap of these two results (yellow). The areas closest to cluster maxima are marked in the same colors as the contrast results that achieved significance (red or green) or both (yellow). The clusters are shown overlaid on the ICBM152 WM template, so that slices correspond to (or close to) z coordinate (from left to right: 6, 9, 18, 30, and 51 mm, MNI space) of maximal voxel values within significant clusters. The letters A, B, C, D, and F indicate the corresponding cluster peak location between the figure and Table 1. A: right occipital WM; B: right anterior internal capsule; C: splenium; D: left anterior frontal subgyral WM; E: left frontal subgyral WM; F: left premotor subgyral WM; LH: left hemisphere; RH; right hemisphere.

Regarding DTI data, between-group comparisons did not reveal any differences between questionnaire-based groups, whereas for performance-based groups, the High > Low risk-taking group contrast revealed significant FA differences. Similarly, only positive correlation between the total number of Go responses (i.e., risky decisions) and FA were found. Because both between-group and covariate analyses were based on the same measure (number of Go responses), their results expectedly demonstrated partial overlap (Fig. 1, Table 1). In particular, the right occipital WM area (Fig. 1, A) was highly significant in both analyses. Another area of overlapping results was located beneath the left prefrontal cortex (BA 9, 10, 46; Fig. 1, D), although with only a bordering significance (p = 0.051) for the between-group analysis. Other clusters were obtained only in one of the analyses. Thus, the High > Low risk-taking group difference was most prominent in the splenium of the corpus callosum (Fig. 1, C). The same contrast also revealed a left frontal subgyral WM difference next to BA 9 and 46 (Fig. 1, E) and a cluster in the anterior internal capsule next to the right thalamus (Fig. 1, B). Positive correlation between FA and number of Go responses were found in the anterior frontal region in the vicinity to BA 10 and 32, as well as under the premotor cortex (BA 6; Fig. 1, F).

**Table 1:** Results of between-group comparisons and regression analysis of the total number of Go responses (i.e., risky decisions) using FA data.

Contrast	Structure	Hemi-phere	P_FWEcorr_ cluster value	Number of voxels	Peak voxel Z value	Peak voxel MNI coordinates		
						x	y	z
High vs. Low risk-taking groups	Posterior thalamic & optic radiation (A)	R	<0.001	756	4.87	33	−69	6
	Frontal subgyral WM (E)	L	0.049	80	4.86	−27	30	30
	Splenium of corpus callosum (C)	L	0.003	1684	4.38	−6	−36	18
	Anterior limb of internal capsule (B)	R	0.022	222	4.00	6	−5	9
	Anterior frontal subgyral WM	L	0.051*	127	3.84	−15	50	21
Total number of Go responses	Posterior thalamic & optic radiation (A)	R	0.012	345	4.48	30	−69	6
	Premotor subgyral white matter (F)	L	0.029	95	4.34	−17	−3	51
	Frontal subgyral WM (D)	L	0.003	298	4.24	−17	48	16

Results surviving a FWE-corrected p<0.05 cluster extent threshold are reported, and alphabets in parentheses to designate corresponding areas in Fig. 1.

*non-significant trend.

No significant correlation between the index of social influence and either GM volume or FA was found.

## Discussion

We discriminated high and low risk-taking adolescents by assessing their propensity for risky behavior and vulnerability to peer influence with personality tests, and compared structural differences in gray and WM of the brain with VBM and DTI, respectively. We also verified their actual risk-taking behavior using a simulated driving task including a peer competition condition, but there was a discrepancy between the self-reported personality test results and task performance. Thus, we also evaluated differences in the brain structure according to task performance using Go response rates (risky decisions).

Comparison between high and low risk-taking adolescents according to personality test results revealed no significant difference in GM volume and WM integrity. However, comparison according to actual risk-taking behavior during task performance revealed significantly higher WM integrity in the high risk-taking group. These results are consistent with previous findings that engaging in risky behavior was associated with more mature WM [30], and greater impulse control was associated with lower WM integrity [29]. These findings are contrary to conventional wisdom that increased risky behavior during adolescence is attributed to the immature brain.

These findings suggest that adolescents who engage in more dangerous (e.g., more adult-like or mature for their chronological age) behavior may have structurally more mature brain than relatively conservative peers. The maturation of the adolescent brain can be influenced by both environmental experience and genetic factors. Thus, adolescents who engage in exploratory and risky behavior may gain more experience in various domains, promoting maturation of the brain, while their conservative peers may not have much experience in life and thus have less mature brains. Biologically, precocious development may predispose some adolescents to risky behavior. It has been posited that puberty leads individuals to biological maturity sooner than society permits [43], [44], and engagement in risky behavior is an attempt to bridge this ‘maturity gap’ by demonstrating a certain level of social maturity and autonomy [45].

Higher WM integrity has been observed particularly in the frontal cortex in the present and a previous study [30]. This indicates an important cognitive issue: It takes brains to take risks. Thus, high risk-takers with more mature brain would calculate possible gains and risks of their (risky) behavior for the best, and show flexible behavior depending on the changing situation. One could imagine a successful gambler who makes stable profits while keep taking risks. This kind of deliberate risk-taking can be differentiated from reckless risk-taking due to impulsivity such as acting without thinking. In contrast, low risk-takers might not consider all possible options in their situation deliberately, whether beneficial or risky, and become conservative and less flexible in different situations.

Although previous studies [29], [30] found similar results based on self-report, we could not find any differences between groups based questionnaires. The questionnaire items for the sensation-seeking factor might not be suitable to predict risk-taking behavior in situations like a driving game that does not provide exciting sensations. Personality traits such as impulsivity can be strong predictors of adolescent risk-taking behavior [46]–[48], but when considering different forms of impulsivity, sensation-seeking was positively related to executive function, while acting without thinking was related negatively [49]. Thus, acting without thinking would be more strongly related to (at least reckless) risk-taking behavior than sensation-seeking we considered. Here, one could think that those who scored high for sensation-seeking are supposed to have better executive function and more mature brain structure, but it was not the case in the present study and has to be resolved. Nevertheless, the significant results based on actual risk-taking behavior provide stronger evidence for the relationship between adolescent risky behavior and the brain structure.

Increased risky behavior during adolescence, especially in the presence of peers, is thought to imply weak prefrontal cognitive control over behavior as compared with a more rapidly developing subcortical motivation system (e.g., [17]). However, the positive correlation between sensation-seeking and intelligence [50], or working memory [49] suggests that those who exhibit stronger sensation-seeking drives are no less able to exert executive control over their behavior [51]. The positive correlation between WM maturity and risky behavior in the present and previous studies [29], [30] also supports this view.

As a limitation of the study, the sample was limited to 18–19 years old due to their relevance to driving behavior (i.e., eligible to drive), which can only capture a very limited range of the dynamic changes that occur across adolescence. The findings were also based on individual behavioral differences rather than developmental differences in age or puberty stages. The lack of developmental analyses can limit the interpretation of the data in the context of adolescent development, for instance, without comparing developmental differences between groups, the differences in WM integrity cannot be solely attributed to brain maturation but also to individual differences of the same age group or developmental stage. Finally, given the small sample size (n = 13–16 in each group), the lack of differences in GM volume or between questionnaire-based groups might be due to a lack of statistical power. Thus, further investigation is needed with a larger sample covering a wide age range and direct developmental comparisons.
